# Useful And Marketable, New And Traditional

**DOI:** 10.4103/0973-1229.32154

**Published:** 2007

In this section we look into the difference between useful and marketable entities, how industry funds new and not traditional therapies and what impact this has on biomedical advance and patient care.

## Useful And Marketable

Focus on the marketability rather than usefulness of products is an area of concern. There is an inevitable slant to produce not necessarily useful but marketable products that ensure the profitability of industry and the research grants outflow to academia.“*…as a society, we need to think about the consequences, for health research as a whole, of the strong focus on academic—industry partnerships to produce marketable products”* (Baird, 2003). A disturbing but very relevant finding in this connection is that drugs which can be called “substantial improvements” over available treatments is only, mark the finding, a measly 6% (Patented Medicine Prices Review Board annual reports [1988-2001]). So it is understandable that companies want to control data collection and dissemination (Baird, 2003).

Which means all the hype touted around ‘new’, ‘improved’ and fancy explanations of phamacokinetics in industry-churned glossy monographs and brochures, is so much hot air. For all the money poured in by industry and research carried out by academia and all the reams over clinical drug trials published all over the world, just 6% is any worthwhile improvement. The rest is, well, trash, suitably packaged to hold attention and boost sales and make for seminar material for CME speakers and industry-sponsored trips all over the country and abroad.

Also, it makes eminent sense for ‘reported outcomes of sponsored trials to highly favour the manufacturer's product’ (Procyshyn *et al.*, 2004).

So we know why it makes sound business sense to control both the data collected and the data disseminated, for industry must at all times appear to forward research and always retain control over what is published, never mind it makes very little material difference to patient welfare. In any case, that is at best an embarrassing reality necessary to negotiate and at worst the cause of so much industry-academia straining of relations on ethical grounds. If only this irksome necessity did not exist how much better for every one around!

How much more will academia need to remove its blinkers?

## Funding For New, Not Traditional, Therapies

We need mention here an interesting relatively old study for its findings. Because of concerns about conflicts of interest and published research, Davidson_(1986)_analyzed 107 controlled clinical trials. Studies were classified as favouring either a new or a traditional therapy and as being supported by a pharmaceutical manufacturer or as being generally supported. Seventy-one per cent of the trials favoured new therapies; pharmaceutical firms funded 43% of these. Of the 31 trials favouring traditional therapy, only four (13%) were supported by a pharmaceutical firm (Davidson, 1986).

The writing on the wall is clear, is it not? Industry supports new therapies, not traditional therapy, irrespective of what is effective. Whatever traditional therapy is supported is also most probably because the industry concerned has a product with a big stake there, which has remained a ‘gold standard’ or which that player thinks has still some ‘juice’ left.

There was a statistically significant association between the source of funding and the outcome of the study (P = 0.002). Few trials supported by pharmaceutical manufacturers favoured traditional therapy; some reasons for this finding may include selection of drugs likely to be proven efficacious, Type II errors (false-negative studies) and fear of discontinuation of funding should such studies be submitted (Davidson, 1986).

That few manufacturers favoured traditional therapy is for obvious reasons. Reasons like selection mistakes or false negative studies are given just to complete the list. The major reason is the fear of discontinuation of funding should such findings be published, for it undermines profitability that results from new product launches and the hype and marketing brouhaha that justifies it. That is the reason major research players desist from drug research that involves old product reconfirmations, are involved rather in old product rejection; are not involved in publication of negative studies of their pet research products and are actively engaged in new drug research and promotion. Not for nothing does systematic review and meta-analysis of evidence find strong and consistent evidence that industry-sponsored research tends to draw pro-industry conclusions’ (Bekelman *et al.*, 2003) and ‘reported outcomes of sponsored trials tend to highly favour the manufacturer’s product’ (Procyshyn *et al.,* 2004).

So what if traditional therapies may still be useful? So what if important clinical information may be lost if negative studies are not published? So what if what appears new today is only to be played down as outdated tomorrow? So what if researchers and academicians know that only pro-industry research results tend to get published? That's the name of the game, honey
